# Implementation of different relationship estimate methodologies in breeding value prediction in kiwiberry (*Actinidia arguta*)

**DOI:** 10.1007/s11032-023-01419-8

**Published:** 2023-10-18

**Authors:** Daniel Mertten, Samantha Baldwin, Canhong H. Cheng, John McCallum, Susan Thomson, David T. Ashton, Catherine M. McKenzie, Michael Lenhard, Paul M. Datson

**Affiliations:** 1 The New Zealand Institute for Plant and Food Research Ltd (PFR), Auckland, 1142 New Zealand; 2 Institute for Biochemistry and Biology, University of Potsdam, 14476 Potsdam-Golm, Germany; 3 Plant and Food Research, Lincoln, 7608 New Zealand; 4 Kiwifruit Breeding Centre, Auckland, 1142 New Zealand; 5 Plant and Food Research, Nelson, 7010 New Zealand; 6 Plant and Food Research, Te Puke, 3182 New Zealand

**Keywords:** Best linear unbiased prediction, Autopolyploid, *Actinidia arguta*, Genomic selection, Accuracy, Cross-validation

## Abstract

In dioecious crops such as *Actinidia arguta* (kiwiberries), some of the main challenges when breeding for fruit characteristics are the selection of potential male parents and the long juvenile period. Currently, breeding values of male parents are estimated through progeny tests, which makes the breeding of new kiwiberry cultivars time-consuming and costly. The application of best linear unbiased prediction (BLUP) would allow direct estimation of sex-related traits and speed up kiwiberry breeding. In this study, we used a linear mixed model approach to estimate narrow sense heritability for one vine-related trait and five fruit-related traits for two incomplete factorial crossing designs. We obtained BLUPs for all genotypes, taking into consideration whether the relationship was pedigree-based or marker-based. Owing to the high cost of genome sequencing, it is important to understand the effects of different sources of relationship matrices on estimating breeding values across a breeding population. Because of the increasing implementation of genomic selection in crop breeding, we compared the effects of incorporating different sources of information in building relationship matrices and ploidy levels on the accuracy of BLUPs’ heritability and predictive ability. As kiwiberries are autotetraploids, multivalent chromosome formation and occasionally double reduction can occur during meiosis, and this can affect the accuracy of prediction. This study innovates the breeding programme of autotetraploid kiwiberries. We demonstrate that the accuracy of BLUPs of male siblings, without phenotypic observations, strongly improved when a tetraploid marker-based relationship matrix was used rather than parental BLUPs and female siblings with phenotypic observations.

**Supplementary Information:**

The online version contains supplementary material available at 10.1007/s11032-023-01419-8.

## Introduction

Successful plant breeding is the art of identifying and selecting potential parents with desirable traits and exceptional performance for the next round of crossing from within a variable population. Modern plant breeding can utilise further information, such as genomics and new statistical analysis tools, to improve parental selection.

Variation in any trait is due to genetic, environmental and other factors (such as maternal effects and crop management tools). Selection on a trait requires that much of the variation in the trait be due to segregating heritable genetic factors rather than due to the environment. To estimate the genetic effects, statistical methodologies that estimate best linear unbiased predictions (BLUPs) have been developed to estimate variance components and predict breeding values (Patterson and Thompson 1971; Henderson 1974). With improvements in computational power and computing techniques, these approaches have been modified and improved to increase their accuracy in predicting breeding values. Incorporating pedigree information and environmental effects into these statistical methods increases the accuracy of genetic analysis of quantitative traits by eliminating some of the bias linked to the sharing of genes among related individuals and has led to a faster genetic gain in animal- as well crop-breeding programmes (Kennedy and Sorenson 1988; Kennedy et al. 1988). The covariance describing kinship among individuals is represented by the additive relationship matrix (*A*) or numerator relationship matrix (NRM). Rules for calculating *A* have been developed for diploid animal species such as livestock, where gametes carry only one of the two alleles (Henderson 1976). Algorithms have been developed to calculate an additive relationship matrix (*A*) and its inverse (*A*^*−1*^) in diploid species, mainly for animal breeding. In diploid species, it is assumed that gametes cannot carry two or more alleles that are identical by descent (IBD) because of meiotic reduction division. In autopolyploids, where non-preferential pairing of chromosome occurs, such an assumption cannot be made. The *A* matrix is the probability that an allele is identical by descent (kinship coefficient) among individuals, multiplied by two for diploids, by four for tetraploids, by six for hexaploids and so forth (Gallais 2003; Kerr et al. 2012).

In many plant species, the estimation of breeding values is confounded by polyploidy. Whole genome duplication is a common event in angiosperms (Soltis et al. 2004, 2015; Wood et al. 2009; Baduel et al. 2018). Different forms of polyploids are defined by the number of multiple coexisting chromosome sets and the pairing pattern of chromosome inheritance. The two extreme forms are auto- or allopolyploidy, but a mixed form of both can also be found (allo-autopolyploidy). Autopolyploids result from genome duplication or the combination of two very closely related species and show non-preferential chromosome pairing between their homologous chromosomes during meiosis. By contrast, allopolyploids result from the combination of chromosome sets from two or more distantly related species and show preferential chromosome pairing behaviour during meiosis (Sears 1976; Soltis and Soltis 1999; Comai 2005; Soltis et al. 2007). Because of non-preferential chromosome pairing, it has been thought that autopolyploids show a high frequency of multivalent chromosome formations. However, in some autopolyploid crops, including blueberry, kiwifruit and potato, almost exclusively bivalent chromosome formation with occasionally (< 10%) multivalent formation has been observed (Soltis et al. 1993; Qu et al. 1998; Fjellstrom et al. 2001; Wu et al. 2014; Choudhary et al. 2020). Through multivalent chromosome formations, double reduction can occur during meiosis, resulting in sister-chromatids segregating into the same gametes (Bradshaw 2007; Bourke et al. 2015; Muthoni et al. 2015).

When dealing with autopolyploid and pedigree-based relationship information, bias in heritability estimation and breeding value prediction can occur if double reduction is ignored. Double reduction affects the inbreeding rate of a breeding population and therefore the kinship between individuals. Studies in blueberries and potatoes revealed correlations between double reductions and the genome locations of quantitative trait loci (Bourke et al. 2015).

Polyploidy is an important consideration for breeding of kiwifruit (*Actinidia*). Several species and hybrids of *Actinidia* have been introduced into cultivation, the main two being *Actinidia chinensis* (Planch.) var. *chinensis* and *Actinidia chinensis* var. *deliciosa* (A. Chev.) A. Chev. (Huang and Ferguson 2007; Datson et al. 2017). Studies on different *Actinidia* species have revealed non-preferential chromosome pairing in natural and induced polyploidy selections. Non-preferential chromosome pairing occurs during meiosis when chromosomes pair with more than one potential homologue partner. Both natural and induced polyploids in kiwifruit can form multivalent chromosome formations (Mertten et al. 2012; Wu et al. 2014). This finding suggests an adjustment of NRM to polyploidy, and double reduction (ω) should be considered in the breeding strategy of *Actinidia* spp. and other crops that include true autopolyploids with occasional double reduction (Haynes and Douches 1993; Kerr et al. 2012; Choudhary et al. 2020).

Tetraploid *Actinidia arguta* (Sieb. et Zucc.) Planch. ex Miq. var. *arguta* (2*n* = 4*x* = 116) (kiwiberry) is one of the species recently introduced into cultivation. Kiwiberries produce small fruit with a soft, hairless, edible skin. Like other *Actinidia* species, kiwiberries are usually dioecious, with staminate flowers on male vines and pistillate flowers on female vines. Female flowers fully develop ovaries, styles, stigmata and stamens but produce non-viable, empty pollen. Male flowers produce viable pollen but rudimentary female organs lacking ovules that are able to develop into fruit (Rizet 1945; Schmid 1978; White 1990).

The current breeding approach for kiwifruit species, such as *A. arguta*, parallel approaches employed in animal breeding. This methodology entails the selection of genotypes using techniques like single-seed descent, accompanied by the utilisation of pedigree records to preserve critical relationship information. Consequently, genotypes displaying desirable trait performances are carefully chosen and clonally propagated for subsequent commercial cultivation.

Owing to the sex linkage of some desired quantitative traits in dioecious crop breeding, it is not possible to select the superior individuals of genotypes within a cross when phenotype observations cannot be made, e.g., the breeding values of fruit characteristics in male genotypes within the same cross are estimated as family means and cannot be distinguished on an individual level. Thus, there is a need to find methods that enable individual estimation of a trait-value for a non-expressed trait. In particular, the selection of male parents requires progeny testing, as they do not provide phenotypic information on their genetic background, for the breeder. Recently, genomic methods have been developed to enable this prediction (Testolin 2011; Datson et al. 2017; Cheng et al. 2019). In polyploids with their multiple homologous chromosome sets, allele dosage information is crucial to estimating marker-based additive variance–covariance relationships between individuals to predict breeding values. To date, there is no publication addressing the application of genomically estimated breeding values (GEBV) to breeding of autotetraploid kiwiberries.

To explore the effects of incorporating probabilistic versus realised relationship matrices into a linear mixed model equation for commercially important fruit quality traits and vine characteristics, we modified the equation through the application of different types of relationship matrices (using pedigree or genomic information) and varying the complexity of assumptions of chromosome inheritance. The effects of these modifications on the breeding value estimates for parental generations, female progenies with trait records and male progenies with no records, are compared.

## Materials and methods

### Plant population and phenotyping

A seedling population of tetraploid *A. arguta*, consisting of two incomplete factorial crossing designs (Supplementary Table 1), was generated within the parental breeding programme at The New Zealand Institute for Plant and Food Research Limited (PFR). In 2014, 1791 seedlings from 50 crosses were planted at the PFR Motueka Research Centre (41°50′ S; 172°58′ E). A minimum of 20 randomly selected seedlings with a mix of males and females per cross was planted in groups of seven with replication at cross-level in the field trial. Plants were separated by distances of 0.5 m within a row and 3.0 m between rows. Seedlings were grown on a pergola support system, the most common production system used in New Zealand. Upon plant establishment, the observed number of seedlings per cross exhibited a range spanning from a minimum of 2 seedlings to a maximum of 80 seedlings. Notably, only 3 of the 50 crosses yielded fewer than 10 seedlings, whereas 5 crosses yielded a number of 40 seedlings. Overall, there were 36 progeny per cross on average, with a median of 39 progeny for each cross. Plants were established in the field for 2 years, after which fruiting vines were assessed. Two canes from the current growing season were trained horizontally during summer and remained after winter pruning for vine assessments. The numbers of progeny within each cross varied, and phenotype data of some individuals were missing in some years, making the phenotypic data incomplete.

One vine characteristic (fruit load) and five fruit characteristics (fruit weight, dry matter, ripe soluble solids content, fruit circularity crosswise and lengthwise) were assessed for this study. During the 5-year trial, fruit load was recorded in 2017 and 2018. Fruit load was scored from 0 to 9 (Supplementary Table 2), but category zero individuals, with no fruit, were not included in this study. The assessment of fruit load in female vines followed a scoring system based on the number of fruits they bore. Vines with a fruit count of up to 4 received a score of 0.5. Those with up to 10 fruits were assigned a score of 1, while vines carrying up to 30 fruits garnered a score of 2. As the fruit load increased, the scoring correspondingly escalated: vines with up to 60 fruits achieved a score of 3, those with up to 100 fruits were rated at 4 and vines containing up to 200, 300, and 400 fruits received scores of 5, 6 and 7, respectively. Vines that developed up to 500 fruits were designated a score of 8, whereas vines shouldering more than 500 fruits attained the highest score of 9. Fruit assessments were performed when fruit maturity was indicated by > 90% of seeds being black. Fruit weight (g), recorded from 2017 to 2019, was the mean of 30 randomly picked fruit across each vine. Dry matter percentage (DM) was recorded from 2017 to 2019. Three representative fruits were sampled randomly, and a cross-sectional slice of 2–5 mm was cut for DM calculation (Fenton and Kennedy 1998). Ten fruits were sampled from harvest and kept at 1 °C for 14 days, followed by 1 day at room temperature to ripen. Ripe soluble solids content (SSC) of three sampled ripe fruits was measured in 2018 and 2019 using a digital pocket refractometer (ATAGO®). Six fruits, when available on the vine, were taken for measuring fruit circularity in 2019 and 2020. Three fruits were cut in half lengthwise and placed flesh side up on a black background. From the remaining three fruits, an equatorial 5-mm slice (crosswise) was cut and also placed on black background. The outline of fruit was extracted from images using background thresholding from the OpenCV library. The circularity of the fruit outline was then measured as the proportion overlap between the area of the outline and the area of a circle that was the same total area as the outline and centred on the outline (1 = perfect circle). The trait properties were analysed using the R-package “moments” v. 0.14.1 (R Core Team 2020; Komsta and Novomestky 2022).

### DNA extraction and genotyping

Young leaf tissue was collected in spring, and DNA was extracted by Slipstream Automation (Slipstream Automation, Palmerston North, New Zealand). Final dsDNA concentration was standardised to a quantity of ~ 500 ng per sample and vacuum dried to the requirement of the high throughput targeted resequencing platform Flex-Seq® Ex-L of RAPiD Genomics (RAPiD Genomics Gainesville, FL, USA). Resulting sequence reads were mapped against the *A. chinensis* var. *chinensis* “Russell” reference genome (Tahir et al. 2022). Alignments were generated using BWA-MEM (Li 2013) and SAMtools (Danecek et al. 2021) using default parameters. SNP calling was performed in ANGSD with region selection based on target intervals (Korneliussen et al. 2014). Dosage estimation of tetraploid *A. arguta* x *A. arguta* population and SNP filtering were performed using the R-package “Updog” V2. Dosage genotypes were called for offspring and parental lines using an empirical Bayesian approach (Gerard et al. 2018). A further filtering of SNPs was performed for quality, allele bias (0.5 < bias < 2), over-dispersion (od < 0.02) and sequencing error (seq < 0.01) (R Core Team 2020; Tahir et al. 2020). Genotypes were called under the tetraploid (4*x*) assumption as 0 (AAAA), 1 (AAAB), 2 (AABB), 3 (ABBB) and 4(BBBB). For pseudo-diploid (2*x*) genotyping, all heterozygote genotypes were assumed to be one class and therefore recoded as 0 (AAAA = AA), 1 (AAAB, AABB, ABBB = AB) and 2 (BBBB = BB).

### Linear mixed model and relationship matrices

A linear mixed model (LMM) was used to predict breeding values for a segregating population comprising two incomplete crossing designs:

$$y=\mu +Xb+Za+e$$

where $y$ is a vector of phenotypic values of the analysed trait, $\mu$ is the overall population mean, $b$ is a vector of fixed effect (multiple years of observation) with the incidence matrix $X$, $a$ is the unobserved random effect of genotypes with $a\sim \mathrm{N}(0,\mathbf{G}{\sigma }_{\mathrm{a}}^{2})$ where ${\sigma }_{\mathrm{a}}^{2}$ is the additive variance and $Z$ the incidence matrix of genotypes and $e$ is the random residual effect with $e\sim \mathrm{N}(0,\mathbf{I}{\sigma }_{\mathrm{e}}^{2})$.

Variance components and their standard errors were estimated using ASReml-R software in R (Gilmour et al. 2015; R Core Team 2020). ASReml-R uses restricted maximum-likelihood (REML) methodology, which can be applied to unbalanced crossing designs (Patterson and Thompson 1971). Narrow-sense heritability (${h}_{\mathrm{NS}}^{2}$) on an individual plant basis was estimated for each trait, considering the proportion of additive variance component and total variance component ${\sigma }_{\mathrm{p}}^{2}$ : ${h}_{\mathrm{NS}}^{2}=\frac{{\sigma }_{\mathrm{a}}^{2}}{{\sigma }_{\mathrm{p}}^{2}}$ (Falconer and Mackay 1996).

We considered several different approaches for building the relationship matrices to estimate BLUPs. The effect of pedigree-based and marker-based relationship matrices and the effect of including ploidy-levels and double-reduction coefficients to build the variance–covariance matrices were compared. The R package “AGHmatrix” v. 2.0.4 (Amadeu et al. 2016; R Core Team 2020) was used to build all relationship matrices. The methodologies used in this study are summarised in Table 1

**Table 1:** Model used to estimate breeding values of dioecious *Actinidia arguta* kiwiberry crop. (*) A double reduction coefficient (ω) of 0.01 was also chosen to implement a multivalent chromosome behaviour during meiosis. (**) A minor-allele frequency (maf) of 0.05 was chosen by the visual validation of normality of the residual

Relationship matrix	Model	Ploidy	Reference
Pedigree-based	A2	2x	(Henderson 1976)
	A4	4x	(Kerr et al. 2012)
	A4^ω^	4x*	(Kerr et al. 2012)
Marker-based	G2	2x**	(Yang et al. 2010)
	G4	4x	(VanRaden 2008) adapted by (Ashraf et al. 2016)

### Model comparison and cross-validation

The plant population in this study can be divided into different levels of sub-populations. The core element is a total number of 842 female progeny with phenotype and genotype information used to estimate BLUPs, while 910 male progeny, 31 parents and 11 distantly related ancestors contribute only genotype information but no phenotype information. Because of the lack of developing fruit, 39 seedlings did not contribute any phenotypic information but were included in the genotyping process. Owing to the sex-linkage of fruit traits, only female progeny contributed phenotypic information to the BLUP estimation. Most of the parents used to develop the two factorials had been developed from previous controlled crosses, and pedigree information for each of these was available. The 13 females in the first factorial were selected for their own performance and crossed with two male selections from the germplasm at PFR Motueka. The second factorial comprised 13 male parents, previously selected from their seedling populations based on their overall family means and crossed with two commercial female cultivars (Supplementary Table 1).

**Fig. 1:**
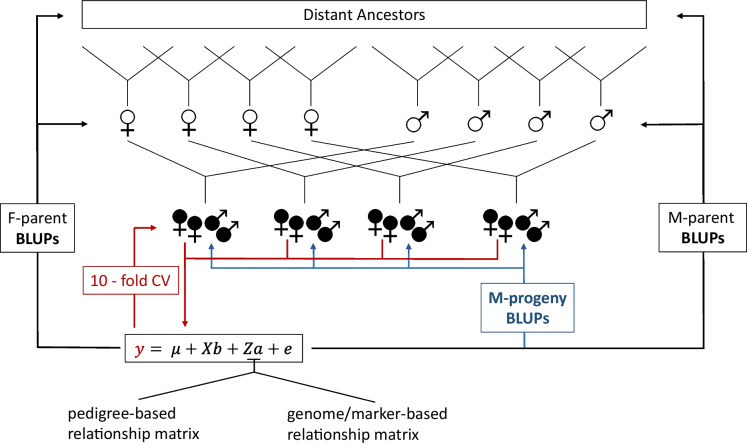
Overview of breeding value predictions for female (F-) and male (M-) *Actinidia arguta* genotypes. From a previous population of 12 crosses (distant ancestors), 13 females, one unrelated additional female and 13 males were selected and used as parents within two incomplete factorial designs, represented by four females ♀ and four males ♂. From the two factorials, 842 female (  ) and 910 male (  ) progeny were selected. Only female progeny were phenotyped and used as response variable ($y$) within the linear mixed model (LMM). The full model was used to predict best linear unbiased predictions (BLUPs) for female and male parents as well as male and female progeny. A tenfold cross-validation (tenfold CV) was set and repeated 10 times to validate the predictive ability for phenotyped female progenies

An overview of breeding value prediction is provided in Fig. 1. A total of 1752 progeny of the two factorial crossing schemes was used. Phenotypic information of female progeny was used to predict breeding values (BLUPs) for both the parental generation and progeny generation under the assumption of a pedigree-based or marker-based relationship matrix. The LMM was validated by applying a tenfold cross-validation scheme to compute different validation criteria. For cross-validation, female progeny with phenotypic observations were assigned randomly into 10 groups. At each validation step, information from one group (validation set) was masked and predicted by the remaining groups (training set). The randomised grouping was repeated 10 times to eliminate structural occurrences in datasets and the population. Individuals without phenotypic observation records (parental and male individuals) were not included in the model validation method (Supplementary Fig. 1). Each group was used only once as a validation set, and the correlation of observation to prediction (predictive ability, $PA$), mean squared error (MSE), regression coefficient of observed phenotypes to their breeding value prediction (bias), variance components and expected genetic gain (EGG) were calculated. The genetic gain was estimated using the following equation: $\Delta G=\frac{1}{2}(PA*{\sigma }_{a}*i)/L$, where $PA$ is the correlation between observed phenotype and prediction, ${\sigma }_{a}$ is the square root of additive variance, $i$ is the selection intensity and $L$ is the length of breeding cycle. We set $i$ and $L$ equal to 1 to be consistent for all models. Because of dioecy, only female progeny were considered.

A LMM including all female progenies (full model) was used to compute the accuracy of breeding value prediction for all tested variance–covariance matrices. Female progeny with phenotypic observations were used to train the model, and BLUPs were estimated for all individuals included in the crossing scheme. Results were grouped into parental generation (with distant ancestors), individuals with observation (female progenies) and no-observations (male progenies).

The accuracy of BLUP estimation was calculated using the definition described by Henderson in 1975: $accuracy= \surd (1-\frac{PEV}{{\sigma }_{a}^{2}{K}_{ii}})$, where PEV is the predicted error variance of the predicted error of breeding values of each individual and ${\sigma }_{a}^{2}$ is the additive variance and ${K}_{ii}$ the diagonal element of variance co-variance matrix with ${K}_{ii}$ = 1 + F, where F is the inbreed coefficient of individual *i*. The calculation of accuracy requires the diagonal elements of the mixed model equation (LHS = left-hand-side), when calculating standard error ($SEP$):

$$\left(\begin{array}{cc}\mathrm{X^{\prime}}\mathrm{X}& \mathrm{X^{\prime}}\mathrm{Z}\\ \mathrm{Z^{\prime}}\mathrm{X}& {\mathrm{Z}}^{\mathrm{^{\prime}}}\mathrm{Z}+{K}^{-1}\uplambda \end{array}\right)\left(\begin{array}{c}\widehat{\mathrm{b}}\\ \widehat{\mathrm{u}}\end{array}\right)=\left(\begin{array}{c}X^{\prime}y\\ Z^{\prime}y\end{array}\right),$$

where ${K}^{-1}$ is equal to the inverse of ${A}^{-1}$ (pedigree-based) or ${G}^{-1}$ (marker-based) relationship matrix and its inverse therefore $\lambda = \frac{{\sigma }_{e}^{2}}{{\sigma }_{u}^{2}}$ (shrinkage factor) and the coefficient matrix, (Henderson 1975 ; Mrode and Thompson 2014)

$$\left(\begin{array}{cc}\mathrm{X^{\prime}}\mathrm{X}& \mathrm{X^{\prime}}\mathrm{Z}\\ \mathrm{Z^{\prime}}\mathrm{X}& {\mathrm{Z}}^{\mathrm{^{\prime}}}\mathrm{Z}+{K}^{-1}\uplambda \end{array}\right)= \left(\begin{array}{cc}{\mathrm{C}}_{ii}& {\mathrm{C}}_{ij}\\ {\mathrm{C}}_{ji}& {\mathrm{C}}_{jj}\end{array}\right) .$$

Calculating PEV, the diagonal elements of inverse of the coefficient matrix are required, as shown by Henderson (1975):

$${\left(\begin{array}{cc}{\mathrm{C}}_{\mathrm{ii}}& {\mathrm{C}}_{\mathrm{ij}}\\ {\mathrm{C}}_{\mathrm{ji}}& {\mathrm{C}}_{\mathrm{jj}}\end{array}\right)}^{-1}= \left(\begin{array}{cc}{\mathrm{C}}^{\mathrm{ii}}& {\mathrm{C}}^{\mathrm{ij}}\\ {\mathrm{C}}^{\mathrm{ji}}& {\mathrm{C}}^{\mathrm{jj}}\end{array}\right)$$

$$PEV= {\mathrm{C}}^{\mathrm{dd}}{\sigma }_{e}^{2},$$

with diagonal element ${\mathrm{C}}^{\mathrm{dd}}$ of the inverse coefficient matrix, or.

$${PEV}_{i}=\left({d}_{i}{\sigma }_{e}^{2}\right),$$

where ${d}_{i}$ is the diagonal element of the inverse of LHS, and ${\sigma }_{e}^{2}$ is the residual variance. For every individual included in relationship matrix, a standard error is calculated (SEP) with the following:

$$SEP= \surd (var\left(a-\widehat{a}\right)= \surd ({d}_{i}{\sigma }_{e}^{2})$$

(Henderson 1975; Mrode and Thompson 2014; Gilmour et al. 2015; Isik et al. 2017). All models and scenarios were compared using Tukey’s honestly significant difference (HSD) multiple comparison, considering independent runs of each “fold” as well as each iteration, implemented in the R-packages “stats” and “multcompView” v. 0.1–8 (Hothorn et al. 2008; R Core Team 2020). Visualisation of data analysis was performed using “ggplot2” V3.3.5, “ggbreak” v. 0.1.1 and “patchwork” v. 1.1.1 in R (Wickham 2016; Pedersen 2020; R Core Team 2020; Xu et al. 2021).

## Results

We assessed five methods for calculating the relationship matrix and breeding values accuracy across different generations. All traits showed continuous distributions with a moderate skewness except for fruit load, which had a skewness that was very close to zero and therefore symmetric. Fruit circularity traits were moderately left-skewed (i.e. skewness values were negative), and fruit traits were fairly to moderately right-skewed (i.e. skewness values were positive) (Table 2).

**Table 2:** Overall trait properties for one *Actinidia arguta* kiwiberry vine trait and five fruit traits of female progeny. Trait properties were averaged over multiple years

Trait	Min	1^st^ Qu	Median	Mean	3^rd^ Qu	Max	Skewness	No. of years
Fruit load	0.50	3.00	5.00	4.46	6.00	9.00	0.08	2
Fruit weight (g)	1.00	6.30	7.73	7.98	9.34	19.73	0.60	3
Dry matter (%)	12.44	18.65	20.59	20.62	22.50	29.39	0.14	3
Ripe soluble solids content (Brix)	10.10	14.50	15.91	16.09	17.70	24.50	0.23	2
Fruit circularity (crosswise)	0.92	0.95	0.96	0.96	0.97	0.99	− 0.42	2
Fruit circularity (lengthwise)	0.92	0.94	0.95	0.94	0.96	0.97	− 0.40	2

A total of 1752 *A. arguta* progeny of 50 crosses were planted and managed under commercial breeding programme conditions for 5 years. Pedigree information across the population and 7259 (G4) or 2660 (G2) genome-wide distributed bi-allelic markers were available for analysing the effects of incorporating different relationship matrices.

For the G2 model, genotypes were classified in two homozygote classes and one heterozygote class under the assumption of re-calling genotypes from tetraploid dosage call to pseudo-diploid. Distribution of allele dosage classes under the assumption of 2*x* and 4 × is shown in Supplementary Fig. 2.

### The effect of relationship matrix on variance component estimation and estimated genetic gain

**Fig. 2:**
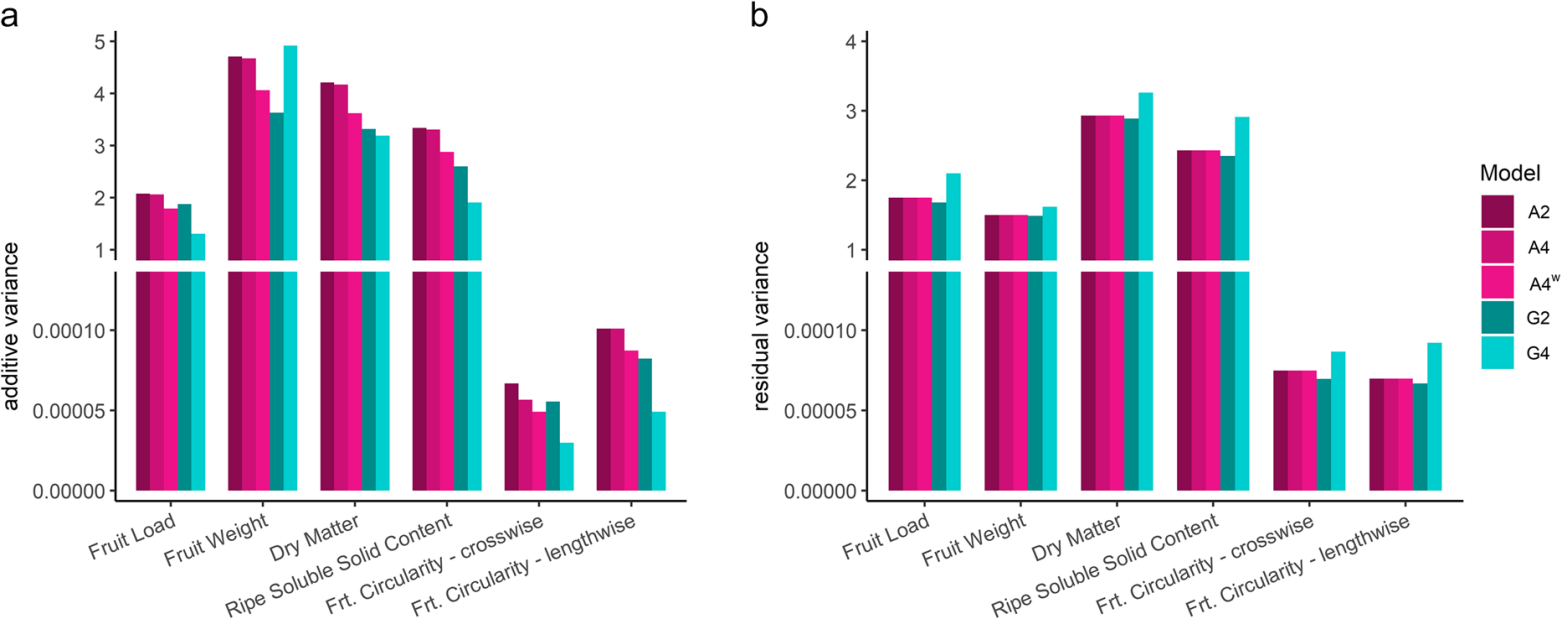
Comparison of variance components. Variance components are compared for a single-vine trait and five fruit traits analysed with a linear mixed model (LMM), considering different relationship matrices, indicated by the letter (A and G), ploidy (2 and 4). In **a**, the comparison of calculated additive variance is displayed, while in **b**, the comparison of residual variance is shown

The genetic parameters of the full model, which includes all progeny with phenotypic information, and the mean over 10 iterations of the tenfold cross-validation model, are summarised in Supplementary Table 3. The impact of the relationship matrix on estimated variance components when employing the full model for all traits is shown in Fig. 2a–b. In the pedigree-based model, the additive variance (Fig. 2a) was consistently higher than that observed under the assumption of marker-based models, across all traits except for fruit weight. There was no difference in residual variance using the full model among the three pedigree-based models (Supplementary Table 3). No significant difference in additive variance between the diploid and tetraploid (pedigree-based) models was observed, except when 10% double reduction was included (Supplementary Table 3). Consequently, narrow-sense heritability, as the ratio of additive to phenotypic variance, showed no significant difference between pedigree-based models under the assumption of disomic (A2) and tetrasomic (A4) inheritance for all traits compared to models including double reduction of marker effects (Supplementary Table 3). Between diploid and tetraploid marker-based methodologies, a significant difference in additive and residual variance was observed (Supplementary Table 3). When G2 was taken into account, the residual variance was lower for all traits, while it was higher considering G4, across all traits (Fig. 2b). In both models (pedigree-based and marker-based), additive variance was very low for both fruit-shape traits compared to fruit-load and fruit-quality traits.

The expected genetic gain (EGG) for each trait and year and overall for the average of multiple years is shown in Supplementary Table 3. Across all traits, no significant difference was observed for diploid and tetraploid probabilistic parametric models. Between the G2 and G4 models, only fruit dry matter content showed no significant differences of overall EGG but showed a significant difference of EGG in 2019. All other traits showed a difference between G2 and G4. The estimate of overall EGG tended to be lower when G4 was used, compared with G2 (Supplementary Table 3).

### The effect of relationship matrix and ploidy level on the accuracy of BLUPs

We investigated the accuracy of predicted BLUPs for all six traits. BLUPs and the corresponding accuracy were estimated for all individuals with or without observations, incorporating different relationship matrix approaches into the LMM equation. The standard error of BLUP estimation, and therefore the accuracy of predicted breeding values of an individual, relies on the available information. Parental BLUPs, and therefore the accuracy of breeding value prediction, depend heavily on phenotypic records of progeny and relatives as well as the number of relatives. However, the accuracy of individuals within the progeny generation depends on the individual performance of those with phenotypic records, or on the family mean for individuals without phenotypic observations.

**Fig. 3:**
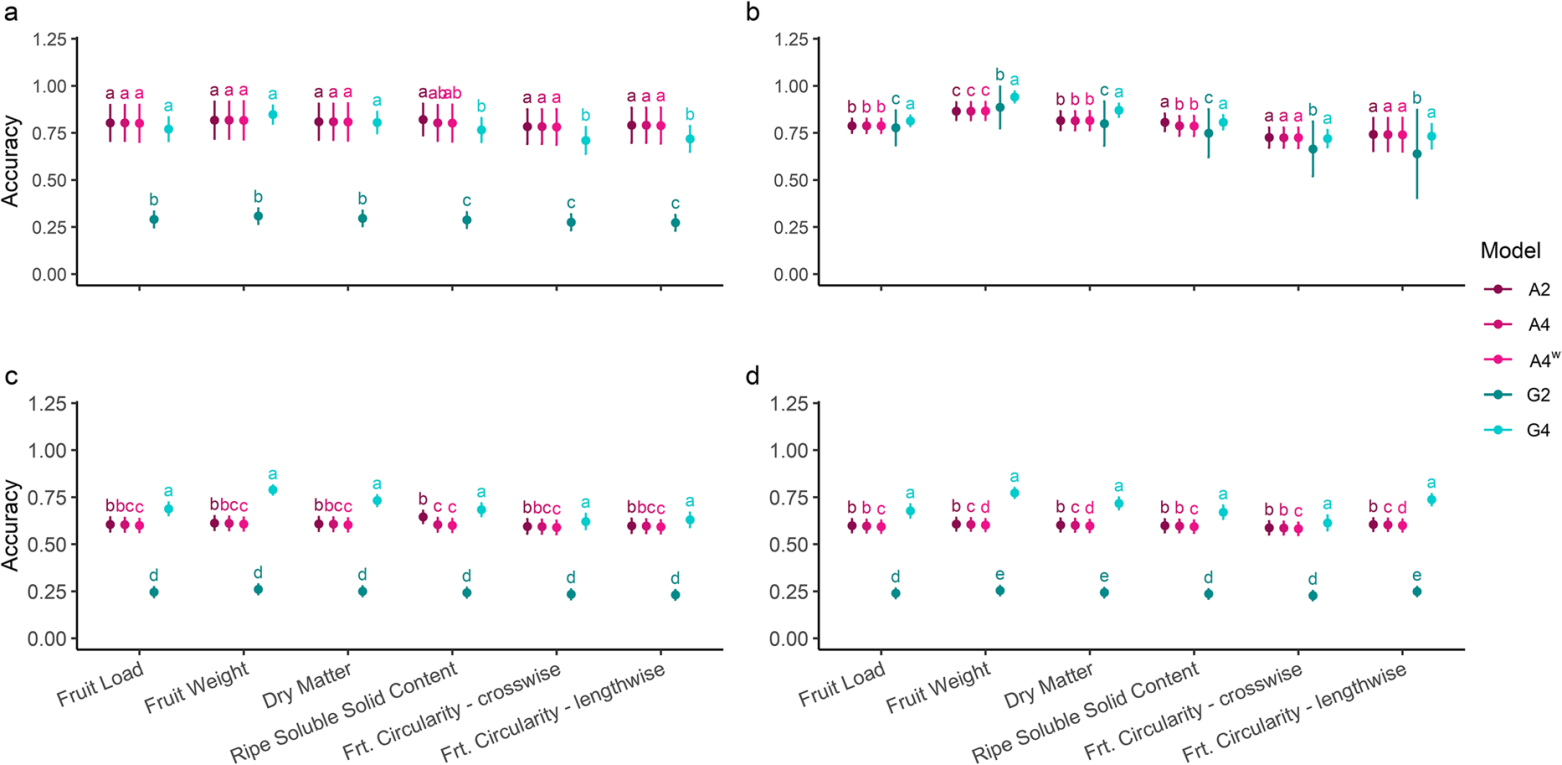
The effects of different relationship matrices (A = pedigree-based and double reduction coefficient *w* = 0.1, G = marker-based) and the effect of considering ploidy level (2 or 4) on the accuracy of best linear unbiased prediction (BLUP) estimation were compared for each *Actinidia arguta* kiwiberry trait and within different sub-populations. Mean accuracy is shown for **a** parental generation, **b** female progeny and **c** male progeny. Plot groups (**a–c**) result from a full-model set; all females with observations are included. Plot group (**d**) is the result of validation sets from a 10 × tenfold cross-validation methodology. Letters are from Tukey’s HSD test; models with the same letter within a trait are not significantly different at a significance level of 0.05

In this study, female progeny with observations and parents without phenotypic observations showed similar high accuracy of breeding value predictions. Within the parental generation, no significant differences were observed when using different pedigree-based relationship matrices. For all traits, the accuracy of prediction was significantly lower under the assumption of pseudo-diploidy of the marker-based relationship, whereas tetraploid genetic marker methodology showed no differences from pedigree-based relationship methodologies (Fig. 3a). Including own phenotypic performance for all female progeny, marker-based relationship matrices significantly improved the estimation of accuracy, compared with pedigree-based methodologies (Fig. 3b). No difference was observed between A2 and A4, but including a double reduction coefficient in the LMM reduced the accuracy (Fig. 3b). The highest effect of realised relationship matrix (G4) on the accuracy of BLUP estimation was observed when individuals had no trait records (Fig. 3c–d). All relationship methodologies were compared for male progenies, which do not have trait records (Fig. 3c). The tetraploid G-matrix significantly improved the accuracy of breeding values. The results of male progeny population were compared with the results of the tenfold cross-validation approach, where observations were masked for females in the validation set (Fig. 3d). The sets performed almost identically.

### Model validation and the effect of relationship methodology and ploidy

We investigated the correlation coefficient between mean observations over multiple years and predicted breeding values when observations were masked (validation set). An indicator of inflation/deflation of predicted breeding value variance was explored. A regression coefficient (*β*) of 1.0 (threshold line) indicates no differences in variance between observed phenotypes and predicted breeding values. In comparison with the pedigree-based LMM approach, A2, A4 and A4^*w*^ showed a mean regression coefficient close to *β* = 1.0, indicating a similar variance among predicted breeding values and mean phenotypic observation (Supplementary Fig. 3a). No significant difference between pedigree-based models was observed. Under the assumption of pseudo-diploidy, a bias > 1 for all traits and a significant difference between G2 and other models were observed. Whereas the tetraploid model (G4) showed a bias less than 1.0 (threshold) and a significant difference from other tested models; a higher variance of predicted breeding values was observed compared with the phenotypic observation (Supplementary Fig. 3a).

The correlation between observation and predicted breeding values (predictive ability) for all tested methodologies of calculating relationship matrices was obtained by computing the overall Pearson’s correlation of each validation set. No difference in predictive ability was observed between pedigree-based and pseudo-diploid realised relationships based on the predicted abilities (*PA*) for all studied traits. *PA* of the tetraploid-realised relationship methodology (G4) varied depending on the trait. A significant difference between G4 and the pedigree-based approach was observed for fruit load, whereas no difference between G2 and G4 was observed for dry matter content, ripe soluble solids content, or fruit circularity (Supplementary Fig. 3b).

The quality of model prediction was measured by the mean-squared error (MSE) for each model approach and trait. In general, the MSE was higher under the G4 model assumption compared with other models. The only significant difference between G4 and the other studied models was observed for fruit weight. No significant difference was observed between all three pedigree-based models and between pedigree-based and G2 models (Supplementary Fig. 3c).

## Discussion

LMMs to estimate best linear unbiased predictions (BLUPs) were first developed in animal breeding to estimate additive random individual effects and are now used in plant breeding. An improvement for predicted breeding values and accelerating genetic gain of economically important quantitative traits can be achieved by including the genetic information of individuals (Meuwissen et al. 2001). Genomic selection uses markers distributed across the whole genome to construct an additive relationship matrix directly from the genotypic information and the covariance relationship of breeding values between individuals (Calus 2010). Genomic-based relationships exploit not just genetic information between families but also differentiate the relationship between individuals within a family, whereas a pedigree-based relationship assumes an equal probabilistic relationship within a family through common ancestors. In this study, five approaches to generating relationship matrices were applied to predict breeding values for six economically important, sex-linked traits in kiwiberry.

Validation variables (accuracy, bias, predictive ability and mean squared error) were estimated under the different relationship matrices (A = pedigree-based, G = marker-based) and accounted for ploidy (2 = diploid and 4 = tetraploid). Studies of double reduction during meiosis in natural and induced tetraploid *A. chinensis* populations showed a 10% multivalent chromosome formation during meiosis in induced tetraploids (Wu et al. 2014).

Evidence of marker segregation in autotetraploids showed that the rate of double reduction increases towards the telomere because multivalent and chromosome formations cross-over events are more likely. Nevertheless, double reduction is often ignored and this, therefore, may be one reason for the low rate of genetic improvement in polyploids compared with their diploid counterparts (Bourke et al. 2015; Amadeu et al. 2016). Testing the effect of double reduction in *A. arguta*, a second A4 model with a double reduction coefficient of 10% (*w* = 0.1) was proposed in this study.

### Source of information and relationship matrix methodology

Our study compared the effects of own performance and observation records of relatives on the breeding value predictions using different methodologies to build a relationship matrix. Breeding value is the estimated merit of genotypes, as parental breeding values are estimated by progeny performance; consequently, the accuracy of BLUP estimation is high (Fig. 3a). Since phenotyped individuals and pedigree relationships were the only sources of information to build this model, the accuracy of progeny with observations was high, regardless of which of the three matrices were used (Fig. 3b). Any progeny which lacked phenotypic observations did not contribute information to the prediction model. Breeding values of progeny without phenotypic records were estimated incorporating phenotypic information of siblings and relatives, which is obviously a less accurate BLUP estimation. The same pattern was also observed in the female progeny population when observations were masked (Fig. 3c–d). This finding suggests that the accuracy of estimation heavily relies on own observation records, and there is no sex-linked effect.

Through the use of markers across the whole genome, the genomic-based relationship distinguished the relationship of individuals within families by marker inheritance. Based on marker inheritance, breeding values can be estimated for individuals where no phenotypic information can be made. When genetic markers were used to build a realised relationship matrix, each individual’s own phenotypic performance became less exclusive to predicting breeding values, compared with the linkage between markers and phenotypic observations. In individuals with just genotypic information, the marker-based relationship matrix allows individual breeding values to be estimated. Female progeny were used to train the model regardless of which model was used to predict breeding values; therefore, the accuracy of predicted breeding values for female progeny across all models equates to the accuracy of pedigree-based models (Fig. 3b). The accuracy of breeding value prediction of male progeny (Fig. 3c) and female progeny with masked phenotypic observations (Fig. 3d) is both highly dependent on their relationship to the training population. Between marker-based models, the G4 model significantly improved the accuracy because of the representation of five genotype classes. In contrast, when G2 was used, the heterozygous classes combined into one, resulting in a masked additive genetic effect and therefore less precise breeding value prediction.

Bias is a sufficient indicator of the shrinkage factor (*λ*), the proportion of residual variance to additive genetic variance. The factor lambda (*λ*) is shrinking the distribution of phenotypic observations towards the population mean, which results in a reduced variation in breeding values. A low shrinkage factor results in high variance of predicted breeding values compared with observed variance. Probabilistic relationship matrix-based models tend to have a bias value of around one, indicating similar variance of predicted breeding values and observation. Therefore, the model prediction is more robust for pedigree-based models. Our marker-based relationship matrix model showed significant differences from the pedigree-based models as well as between allele dosages (Supplementary Fig. 3a). This leads us to conclude that there was under- and overestimation of BLUPs compared with pedigree-based models.

There were limited differences in the predictive ability of probabilistic and realised relationship-based models. The correlation of predicted breeding values and phenotype observations was positive for all traits in this research. Individuals (female progeny) within the validation set did not contribute phenotypic information for model development because their observations were masked. Therefore, BLUPs of individuals within the validation set were the predicted family mean, accounted for by phenotyped family members. This can lead to overestimation of BLUPs, whereas models considering realised relationships lead to more precise prediction. Our tested models showed a slightly lower predictive ability for marker-based models, suggesting improvement of the genotyping approach will improve the predictive ability and the mean squared error (Supplementary Fig. 3b–c).

### Ploidy and double reduction coefficient

The effects of ploidy/allele dosage considering tetrasomic inheritance were studied for pedigree-based and marker-based model approaches. For all sub-populations, no significant differences in the accuracy of model breeding value accuracy, bias, predicted ability and mean squared error were observed between the various ploidy levels under the assumption of probabilistic relationship methodology. Using the kinship matrix to estimate the *A-matrix* was originally developed for population studies with varied ploidy levels (Kerr et al. 2012). With uniform ploidy levels, this study showed no significant differences when comparing the model criteria. It is only in polyploid populations where mixed ploidy occurs that consideration of ploidy in kinship estimation between individuals is necessary when a probabilistic-relationship is considered. Only a slightly significant difference of accuracy of prediction was observed here, including the complexity of double reduction, depending on the trait analysed.

Amadeu et al. (2016) showed that the effect of double reduction is cumulative for breeding populations with long histories and therefore more amenable to breeding value prediction. In populations with shallow pedigree histories like for *A. arguta*, the double reduction is less effective for the BLUP estimation, leading to overestimation of variance components.

The accuracy of parental BLUPs and those of other relatives, when no observations are made, depends on the relationship to individuals in the training set (Henderson 1975; Mrode and Thompson 2014). When heterozygote classes of G4 were re-scored to G2, masked additive allele effects resulted, and therefore a significant difference in the prediction accuracy was observed (Fig. 3a, c–d). Within the training set, the prediction accuracy was reduced when observations were recorded, and a diploid marker-based relationship matrix was considered. This suggested a reduction of additive allele effect linked to phenotypic observations (Fig. 3b).

In our study, we observed no effect of ploidy or double reduction coefficient on the validation criteria (bias, predictive ability, mean squared error) for pedigree-based models, which suggests no significant difference in variance estimation. Considering allele dosage in the marker-based relationship, the variance comparison between phenotypic observations and predicted breeding values was significant. The G2 model showed a higher variability in observations than the predicted. On the other hand, G4 predicted a higher variability in BLUPs than observations (Supplementary Fig. 3a). This leads us to conclude that there was underestimated BLUP prediction using G2 and an overestimation when the G4 model was tested.

Limited differences were observed of predictive ability and mean squared error when ploidy or allele dosage were considered (Supplementary Fig. 3b–c). Gemenet et al. (2020) studied the effect of diploid, pseudo-diploid, tetraploid and hexaploid variant calling in potatoes and sweet potatoes. The authors showed that when diploidized genotype data are considered, it is more adequate to call genotype classes directly as diploid rather than re-diploidizing from high ploidy calls. We can confirm that pseudo-diploidizing, already called genotypes, is less reliable. In autopolyploids, estimating heterozygote genotype classes can be challenging. de Bem Oliveira et al. (2019) compared the influence of different relationship matrices originating from various genotype call data. The authors showed similar results in predictive ability when considering pseudo-diploid and tetraploid marker-based relationship matrices, with only minor differences observed. Due to the challenge of estimating heterozygote classes in autopolyploids, it can lead to misclassification and interfere with genomic selection, as shown in different studies (Grandke et al. 2016; Schmitz Carley et al. 2017; Bourke et al. 2018). An alternative genotyping approach was recommended, using continuous genotyping (de Bem Oliveira et al. 2019).

Our results of predictive ability contrast with the accuracy of breeding value prediction, which improved significantly when the tetrasomic inheritance of the marker-based relationship matrix was considered using the predicted error variance. All female progeny with phenotypic observations were grouped into a training and validation set (tenfold cross-validation). A consequence of grouping these small populations made the comparison of model validation less reliable, as suggested by Gurka and Edwards (2011). Further investigation using large breeding populations in dioecious crops is required.

In this study, we have shown the potential of using different variance co-variance relationship methodologies in *A. arguta* breeding programmes. Overall, the results of six traits considered in a marker-based relationship matrix showed a positive correlation of predictions to mean observations, indicating a better representative genetic architecture of genome-wide marker coverage using a multiplex PCR and new generation sequencing combination approach, compared with previous studies of other *Actinidia* species (Datson et al. 2017; Cheng et al. 2019). We were able to differentiate the effects of different relationship methodologies and ploidy to the best linear unbiased prediction in the parental generation and the progeny population, progeny both with and without phenotypic observation. Including the uncertainty of double reduction to the pedigree-based methodology had less effect on the accuracy of prediction. In the context of selecting genotypes within crosses when no phenotypic observations can be made, pedigree-based models have no power to distinguish variation. Marker-based models allow capturing variation between individuals within the same cross (Daetwyler et al. 2013; de Bem Oliveira et al. 2019). In our study, tetraploid marker-based models incorporating allele dosage significantly affected the predicted accuracy, especially in the progeny generation when no phenotypic observations were available, and these improvements were significant. This will reduce the breeding cycle by at least 3 years because no progeny testing of selected males is needed. The estimated 3 years mainly represent the time required for cross establishment before the first observations can be made. Further work including genotype by environmental interactions and non-additive effects could improve the genomic selection models (Endelman et al. 2018; Matias et al. 2019).

### Supplementary Information

Supplementary Fig. 1 10-fold cross-validation methodology. The base population contained *Actinidia arguta* female progeny with observations (red), parental individuals as well as distant ancestors and male progeny without records (blue). Female progeny with observed records were divided randomly into training and validation sets using a 10-fold cross-validation approach. In the validation set, the observations were masked. Progeny with observations were randomly grouped into 10 groups; each was used once as a validation set (light red), whereas nine groups were used to train the model (training set, dark red). Individuals with no phenotypic information were explored using the full model (PNG 335 KB)

High Resolution (TIF 22.7 MB)

Supplementary Fig. 2 Heterozygosity, distribution of *Actinidia arguta* kiwiberry allele dosage classes shown under re-classification for pseudo-diploid (**a**) and tetraploid dosage classification (**b**) (PNG 107 KB)

High Resolution (TIF 50.5 MB)

Supplementary Fig. 3 Validation variables of the 10 x 10-fold cross-validation approach. **a**) regression coefficient of the mean observed *Actinidia arguta* kiwiberry phenotype (multiple years) and predicted breeding values is described as Bias, with a threshold of 1.0 (grey dashed line), when equal variance is observed, (**b**) the correlation of mean observation over multiple years and predicted breeding values (Predictive Ability), and (**c**) the mean squared error (MSE) of the predicted breeding value and mean observation. A Tukey’s HSD test, conducted at a significance level of 0.05, indicates significant differences by the different letter (PNG 440 KB)

Supplementary file2 (DOCX 26.8 KB)

## Data Availability

The datasets generated and/or analysed during the current study are available from the corresponding author on reasonable request.
