# Supplementary figures and images for: Implementation of different relationship estimate methodologies in breeding value prediction in kiwiberry (Actinidia arguta)
Source: Mol Breed. 2023 Oct 18;43(10):75. doi: 10.1007/s11032-023-01419-8 (PMC10584781; doi:10.1007/s11032-023-01419-8)

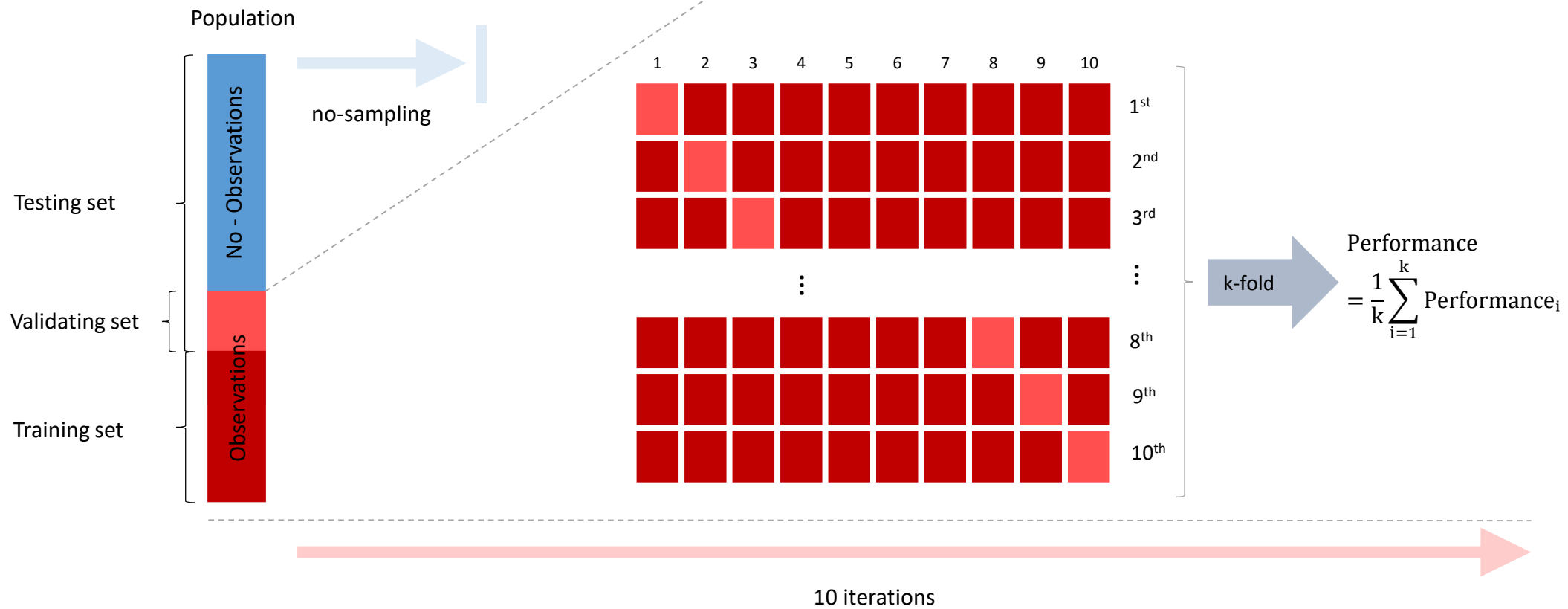

Supplement: Supplementary file 1 — 10-fold cross-validation methodology. The base population contained Actinidia arguta female progeny with observations (red), parental individuals as well as distant ancestors and male progeny without records (blue). Female progeny with observed records were divided randomly into training and validation sets using a 10-fold cross-validation approach. In the validation set, the observations were masked. Progeny with observations were randomly grouped into 10 groups; each was used once as a validation set (light red), whereas nine groups were used to train the model (training set, dark red). Individuals with no phenotypic information were explored using the full model (PNG 335 KB) [file 11032_2023_1419_MOESM1_ESM.pdf]

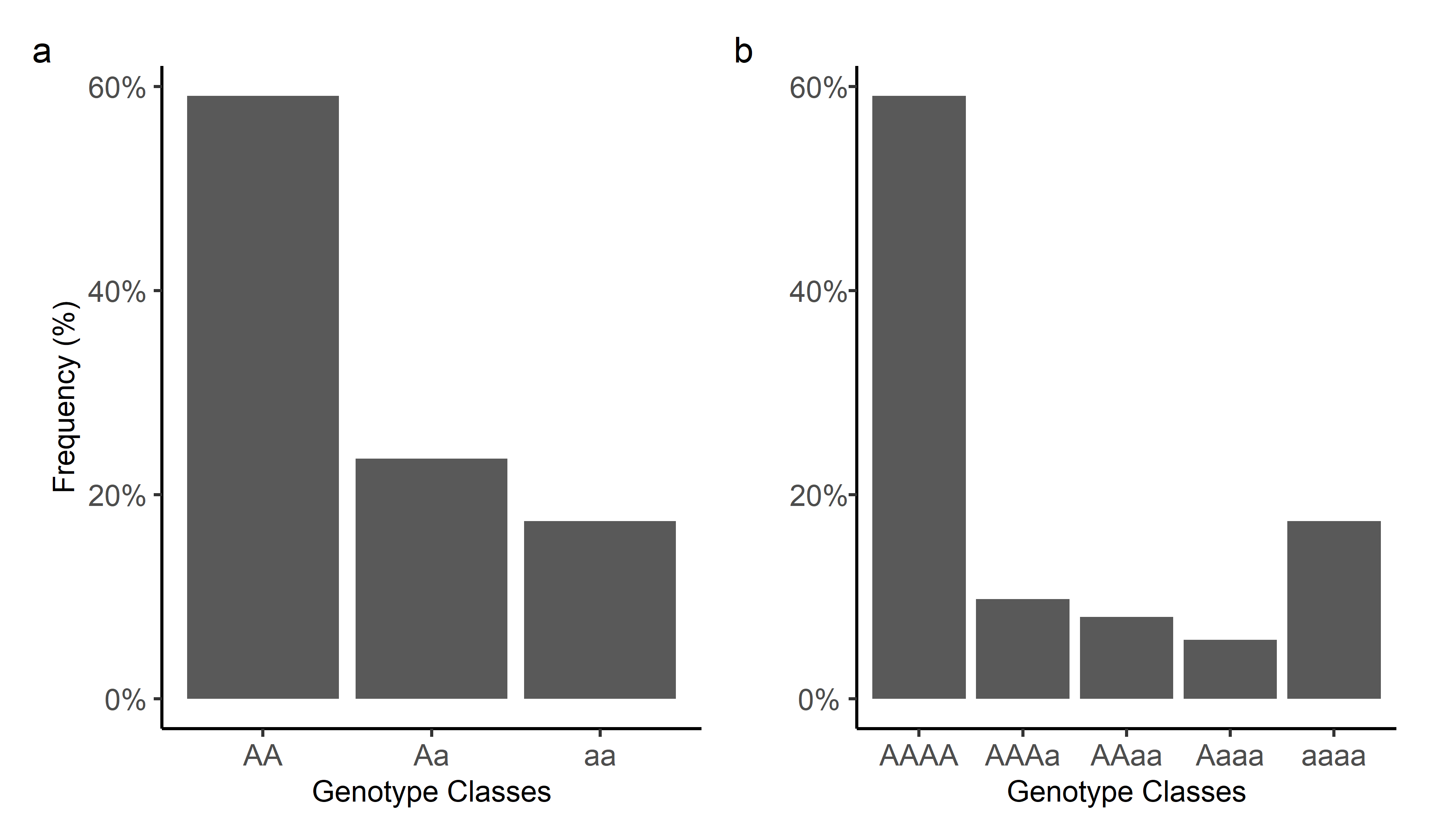

Supplement: Supplementary file 2 — High Resolution (TIF 22.7 MB) [file 11032_2023_1419_MOESM2_ESM.tif]

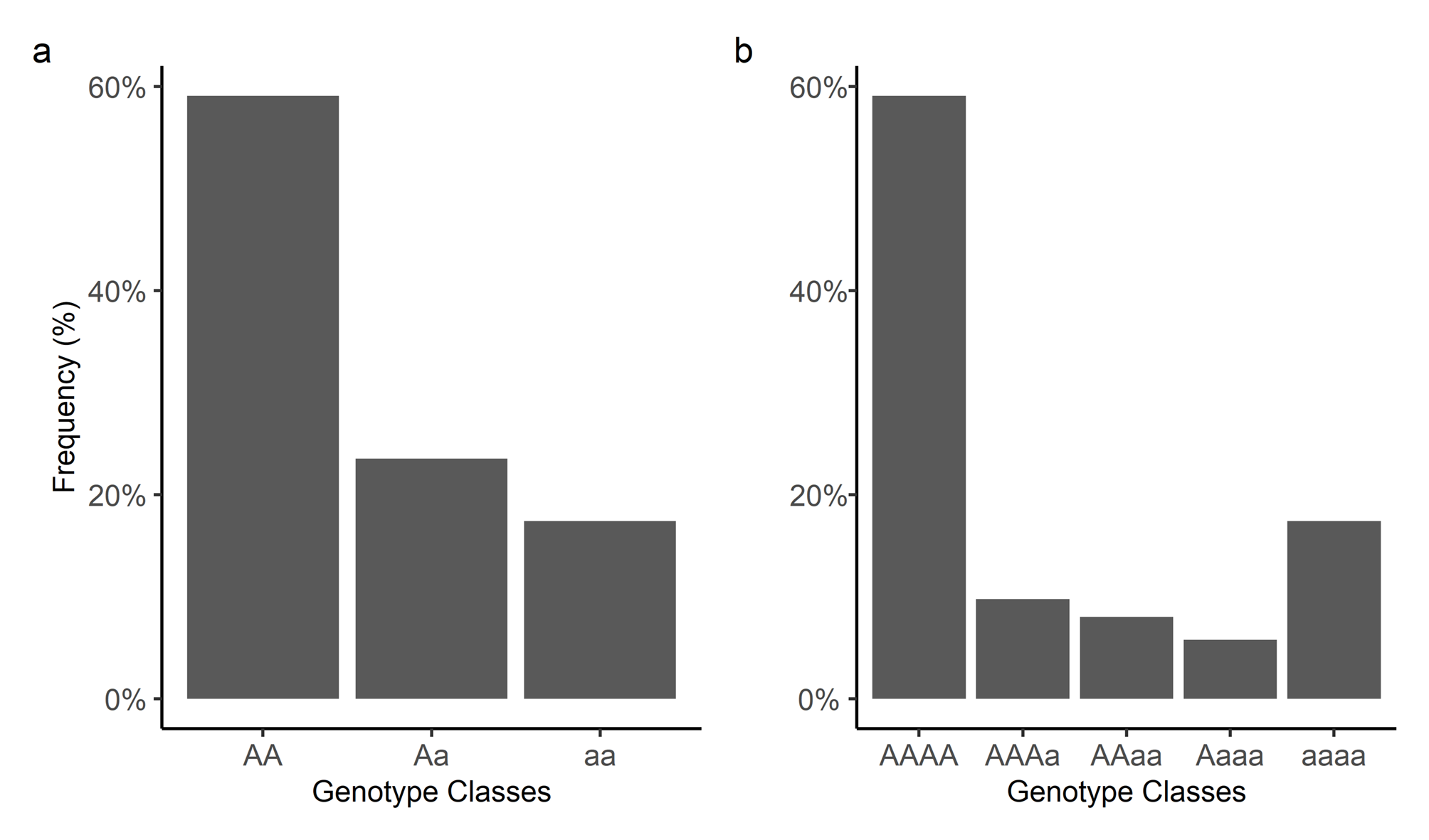

Supplement: Supplementary file 3 — Heterozygosity, distribution of Actinidia arguta kiwiberry allele dosage classes shown under re-classification for pseudo-diploid (a) and tetraploid dosage classification (b) (PNG 107 KB) [file 11032_2023_1419_Fig4_ESM.png]

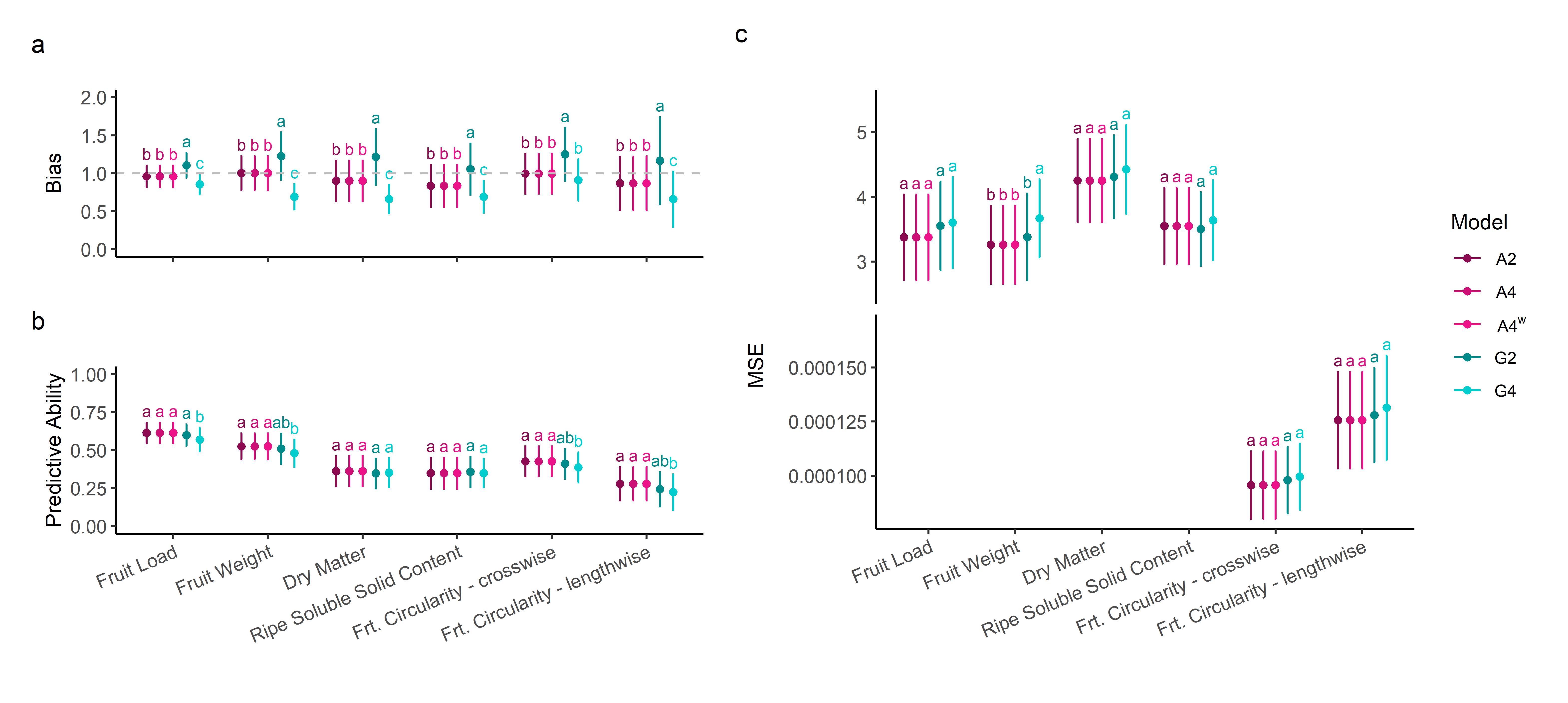

Supplement: Supplementary file 4 — High Resolution (TIF 50.5 MB) [file 11032_2023_1419_MOESM3_ESM.tif]

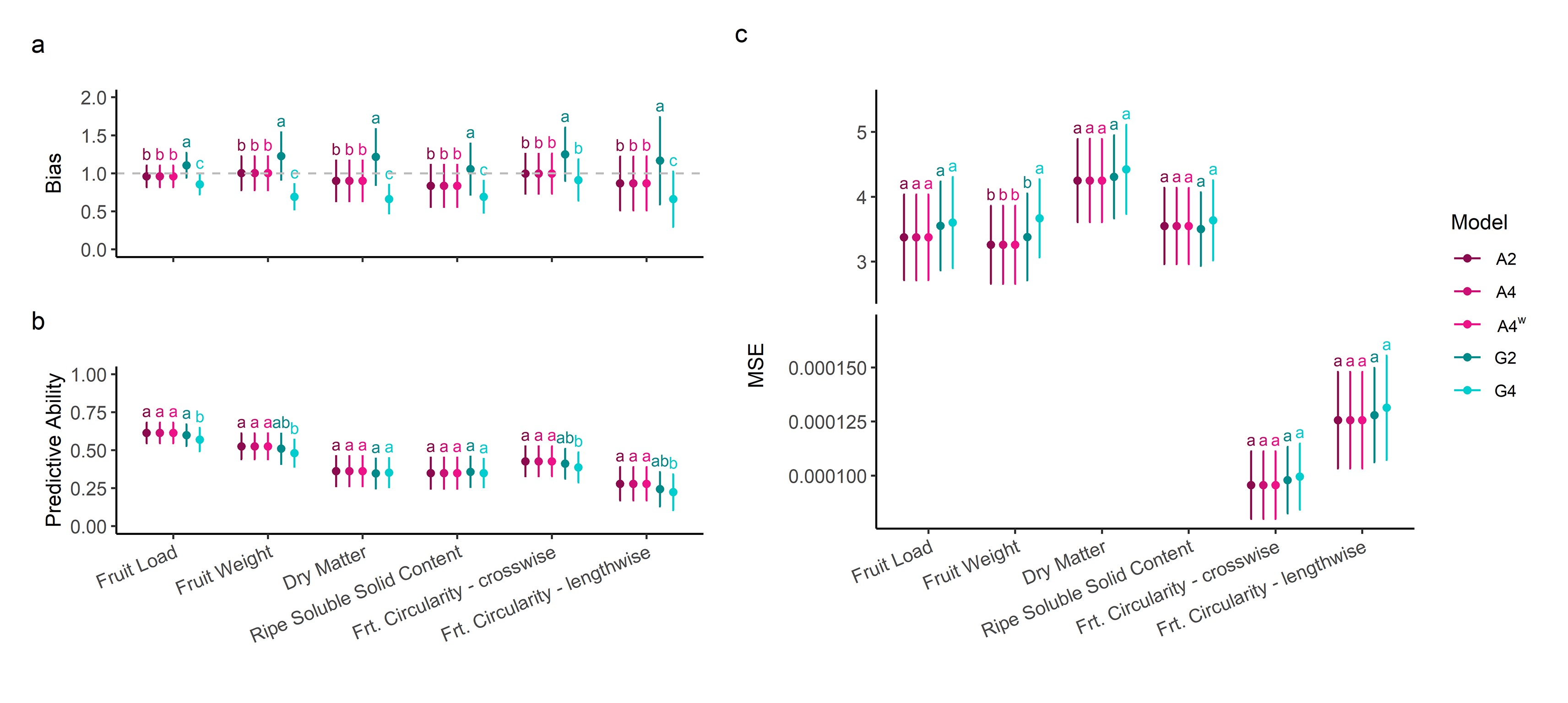

Supplement: Supplementary file 5 — Validation variables of the 10 x 10-fold cross-validation approach. a) regression coefficient of the mean observed Actinidia arguta kiwiberry phenotype (multiple years) and predicted breeding values is described as Bias, with a threshold of 1.0 (grey dashed line), when equal variance is observed, (b) the correlation of mean observation over multiple years and predicted breeding values (Predictive Ability), and (c) the mean squared error (MSE) of the predicted breeding value and mean observation. A Tukey’s HSD test, conducted at a significance level of 0.05, indicates significant differences by the different letter (PNG 440 KB) [file 11032_2023_1419_Fig5_ESM.png]
