# Supplementary material for: Implementation of different relationship estimate methodologies in breeding value prediction in kiwiberry (Actinidia arguta)
Source: Mol Breed. 2023 Oct 18;43(10):75. doi: 10.1007/s11032-023-01419-8 (PMC10584781; doi:10.1007/s11032-023-01419-8)
Supplement: Supplementary file 6 — Supplementary file2 (DOCX 26.8 KB) [file 11032_2023_1419_MOESM4_ESM.docx]

**Implementation of different relationship estimate methodologies in Breeding Value prediction in kiwiberry (*Actinidia arguta*)**

Molecular Breeding

Daniel Mertten*, Samantha Baldwin, Canhong H. Cheng, John McCallum, Susan Thomson, David T. Ashton, Catherine McKenzie, Michael Lenhard, Paul M. Datson

***Corresponding author:**

Daniel Mertten

The New Zealand Institute for Plant and Food Research Ltd (PFR)

Auckland 1142, New Zealand

Email: Daniel.Mertten@plantandfood.co.nz

**Supplementary Table 1** *Actinidia arguta* kiwiberry population structure considering a two factorial crossing scheme with distant ancestral relatives (grey), female parents (red) and male parents (blue). **a)** 13 female and (**b**) male parents are originated from the same cross, indicated by same distant ancestors.

| **a** | |  |  |  |  | **b** |  |  |  |  |
| --- | --- | --- | --- | --- | --- | --- | --- | --- | --- | --- |
| **Distant Ancestors** | |  |  |  |  |  | AA02_01 | AA01_01 | Mother | **Distant Ancestors** |
|  |  |  |  |  |  |  | AA13_01 | AA04_01 | Father |  |
| Mother | Father | **Female Parent** | **Male Parent** | |  | **Male Parent** | **Female Parent** | |  |  |
|  |  |  | **AA05_06** | **AA13_01** |  |  | **‘Hortgem Tahi’** | **‘Hortgem Toru’** |  |  |
| AA06_01 | AA04_02 | **N01.165-12-05** | × | × |  | **N01.161-17-04** | × | × |  |  |
| AA06_01 | AA07_03 | **N01.166-14-04** | × | × |  | **N01.161-04-05** | × | × |  |  |
| AA06_01 | AA13_01 | **N01.166-02-03** | × | × |  | **N01.162-01-03** | × | × |  |  |
| AA06_01 | AA13_01 | **N01.167-13-01** | × | × |  | **N01.162-22-01** | × | × |  |  |
| AA06_01 | AA13_01 | **N01.168-09-05** | × | × |  | **N01.164-06-04** | × |  |  |  |
| AA05_03 | AA04_01 | **N01.166-05-04** | × | × |  | **N01.168-24-01** | × | × |  |  |
| AA05_03 | AA05_06 | **N01.163-01-01** | × | × |  | **N01.161-16-03** | × | × |  |  |
| AA05_03 | AA05_07 | **N01.161-09-02** | × | × |  | **N01.165-20-03** | × |  |  |  |
| AA05_03 | AA07_01 | **N01.167-25-01** | × | × |  | **N01.162-02-04** | × | × |  |  |
| AA06_01 | AA04_01 | **N01.165-11-01** | × | × |  | **-** |  |  |  |  |
| AA06_01 | AA04_01 | **N01.167-11-04** | × | × |  | **-** |  |  |  |  |
| AA06_01 | AA05_07 | **N01.165-09-01** | × | × |  | **-** |  |  |  |  |
| AA05_03 | AA05_06 | **N01.163-01-02** | × | × |  | **-** |  |  |  |  |
| - | - | **AA09_01** | × | × |  | **-** |  |  |  |  |
| AA06_01 | AA07_01 | **-** |  |  |  | **N01.161-02-04** | × | × |  |  |
| AA05_03 | AA04_02 | **-** |  |  |  | **N01.162-19-05** | × | × |  |  |
| AA05_03 | AA04_02 | **-** |  |  |  | **N01.166-02-05** | × |  |  |  |
| AA05_03 | AA07_03 | **-** |  |  |  | **N01.164-09-04** | × |  |  |  |

**Supplementary Table 2** Absolute range of scored *Actinidia arguta* kiwiberry fruit load.

| **Score** | **No. of fruit** |
| --- | --- |
| 0 | 0 |
| 0.5 | 1–4 |
| 1 | 5–10 |
| 2 | 11–30 |
| 3 | 31–60 |
| 4 | 61–100 |
| 5 | 101–200 |
| 6 | 201–300 |
| 7 | 301–400 |
| 8 | 401–500 |
| 9 | 501+ |

**Supplementary Table 3** Estimation of *Actinidia arguta* kiwiberry genetic parameters and expected genetic gain (EGG) for one vine trait and five fruit traits analysed with a linear mixed model (LMM), considering different relationship matrices, indicated by the letter (A and G), ploidy (2 and 4), and significance differences by the letters from Tukey’s HSD test. A double reduction coefficient (*w*) of 10% was incorporated into a second A4 model. A full model, considering all female vines with observations, was included to predict variance components and heritability. The 10-fold cross-validation model was replicated 10 times and each time a new random 10-fold grouping was analysed.

| Trait | Relationship matrix | Full – model | | | 10-fold cross-validation (10x) | | | | | | | |
| --- | --- | --- | --- | --- | --- | --- | --- | --- | --- | --- | --- | --- |
|  |  | $\sigma_{a}^{2}$ | $\sigma_{e}^{2}$ | $h_{\mathrm{NS}}^{2}(\mathrm{SE})$ | $\bar{\sigma_{a}^{2}}$ | $\bar{\sigma_{e}^{2}}$ | $\bar{h_{\mathrm{NS}}^{2}}$ | EGG 2017 | EGG 2018 | EGG 2019 | EGG 2020 | EGG overall |
| Fruit Load | A2 | 2.08 | 1.75 | 0.54 (0.05) | 2.08^a^ | 1.75^b^ | 0.54^a^ | 0.39^a^ | 0.40^a^ | - | - | 0.44^a^ |
|  | A4 | 2.06 | 1.75 | 0.54 (0.05) | 2.06^a^ | 1.75^b^ | 0.54^a^ | 0.39^a^ | 0.40^a^ | - | - | 0.44^a^ |
|  | A4 ^ω^ | 1.79 | 1.75 | 0.51 (0.05) | 1.79^c^ | 1.75^b^ | 0.51^c^ | 0.37^b^ | 0.37^b^ | - | - | 0.41^b^ |
|  | G2 | 1.88 | 1.68 | 0.53 (0.05) | 1.91^b^ | 1.68^c^ | 0.53^b^ | 0.38^ab^ | 0.37^b^ | - | - | 0.41^b^ |
|  | G4 | 1.31 | 2.10 | 0.38 (0.06) | 1.36^d^ | 2.07^a^ | 0.40^d^ | 0.31^c^ | 0.29^c^ | - | - | 0.33^c^ |
| Fruit Weight (g) | A2 | 4.71 | 1.50 | 0.76 (0.02) | 4.71^b^ | 1.50^b^ | 0.76^a^ | 0.58^a^ | 0.52^a^ | 0.50^a^ | - | 0.57^a^ |
|  | A4 | 4.67 | 1.50 | 0.76 (0.02) | 4.67^b^ | 1.50^b^ | 0.76^a^ | 0.58^a^ | 0.52^a^ | 0.48^a^ | - | 0.57^a^ |
|  | A4 ^ω^ | 4.06 | 1.50 | 0.73 (0.03) | 4.06^c^ | 1.50^b^ | 0.73^c^ | 0.54^ab^ | 0.48^ab^ | 0.45^a^ | - | 0.53^b^ |
|  | G2 | 3.63 | 1.49 | 0.71 (0.03) | 3.66^d^ | 1.49^c^ | 0.71^d^ | 0.49^c^ | 0.44^c^ | 0.44^a^ | - | 0.49^c^ |
|  | G4 | 4.92 | 1.62 | 0.75 (0.03) | 4.80^a^ | 1.61^a^ | 0.75^b^ | 0.53^bc^ | 0.47^bc^ | 0.45^a^ | - | 0.53^b^ |
| Dry Matter (%) | A2 | 4.21 | 2.93 | 0.59 (0.05) | 4.21^a^ | 2.96^b^ | 0.59^a^ | 0.40^a^ | 0.33^a^ | 0.41^a^ | - | 0.37^a^ |
|  | A4 | 4.17 | 2.93 | 0.59 (0.05) | 4.18^a^ | 2.96^b^ | 0.59^a^ | 0.40^a^ | 0.33^a^ | 0.41^a^ | - | 0.37^a^ |
|  | A4 ^ω^ | 3.62 | 2.93 | 0.55 (0.05) | 3.63^b^ | 2.96^b^ | 0.55^b^ | 0.37^ab^ | 0.31^ab^ | 0.38^ab^ | - | 0.35^ab^ |
|  | G2 | 3.32 | 2.89 | 0.53 (0.05) | 3.34^c^ | 2.91^c^ | 0.53^c^ | 0.35^bc^ | 0.30^b^ | 0.37^b^ | - | 0.32^b^ |
|  | G4 | 3.19 | 3.26 | 0.50 (0.06) | 3.22^d^ | 3.26^a^ | 0.50^d^ | 0.35^c^ | 0.29^b^ | 0.32^c^ | - | 0.32^b^ |
| Ripe Soluble Solids Content (Brix) | A2 | 3.34 | 2.43 | 0.58 (0.06) | 3.33^a^ | 2.43^b^ | 0.58^a^ | 0.35^a^ | 0.31^a^ | - | - | 0.32^a^ |
|  | A4 | 3.31 | 2.43 | 0.58 (0.06) | 3.30^a^ | 2.43^b^ | 0.58^a^ | 0.35^a^ | 0.31^a^ | - | - | 0.32^a^ |
|  | A4 ^ω^ | 2.88 | 2.43 | 0.54 (0.06) | 2.87^b^ | 2.43^b^ | 0.54^b^ | 0.32^a^ | 0.29^a^ | - | - | 0.30^a^ |
|  | G2 | 2.60 | 2.35 | 0.53 (0.06) | 2.62^c^ | 2.35^c^ | 0.53^c^ | 0.32^a^ | 0.28^a^ | - | - | 0.29^a^ |
|  | G4 | 1.91 | 2.91 | 0.40 (0.07) | 1.97^d^ | 2.87^a^ | 0.41^d^ | 0.26^b^ | 0.24^b^ | - | - | 0.25^b^ |
| Fruit Circularity – crosswise | A2 | 6.68e-05 | 7.49e-05 | 0.43 (0.05) | 5.66e-05^a^ | 7.50e-05^b^ | 0.43^b^ | - | - | 1.45e-03^a^ | 1.58e-03^a^ | 1.61e-03^a^ |
|  | A4 | 5.66e-05 | 7.48e-05 | 0.43 (0.05) | 5.64e-05^a^ | 7.48e-05^b^ | 0.43^b^ | - | - | 1.44e-03^a^ | 1.58e-03^a^ | 1.60e-03^a^ |
|  | A4 ^ω^ | 4.92e-05 | 7.48e-05 | 0.40 (0.05) | 4.91e-05^b^ | 7.48e-05^b^ | 0.40^c^ | - | - | 1.35e-03^a^ | 1.48e-03^a^ | 1.50e-03^a^ |
|  | G2 | 5.55e-05 | 6.96e-05 | 0.44 (0.04) | 5.61e-05^a^ | 6.96e-05^c^ | 0.45^a^ | - | - | 1.42e-03^a^ | 1.50e-03^a^ | 1.54e-03^a^ |
|  | G4 | 2.99e-05 | 8.67e-05 | 0.26 (0.04) | 3.13e-05^c^ | 8.57e-05^a^ | 0.27^d^ | - | - | 9.76e-04^b^ | 1.06e-03^b^ | 1.08e-03^b^ |
| Fruit Circularity – lengthwise | A2 | 1.01e-04 | 6.99e-05 | 0.59 (0.04) | 1.00e-04^a^ | 7.01e-05^b^ | 0.59^a^ | - | - | 1.42e-03^a^ | 1.26e-03^a^ | 1.40e-03^a^ |
|  | A4 | 1.01e-04 | 6.98e-05 | 0.59 (0.04) | 9.98e-05^a^ | 7.00e-05^b^ | 0.59^a^ | - | - | 1.42e-03^a^ | 1.26e-03^a^ | 1.40e-03^a^ |
|  | A4 ^ω^ | 8.74e-05 | 6.98e-05 | 0.56 (0.05) | 8.67e-05^b^ | 6.99e-05^b^ | 0.55^b^ | - | - | 1.32e-03^ab^ | 1.17e-03^ab^ | 1.30e-03^ab^ |
|  | G2 | 8.24e-05 | 6.69e-05 | 0.55 (0.04) | 8.27e-05^c^ | 6.68e-05^c^ | 0.55^b^ | - | - | 1.12e-03^b^ | 9.88e-04^b^ | 1.11e-03^b^ |
|  | G4 | 4.91e-05 | 9.22e-05 | 0.35 (0.05) | 5.10e-05^d^ | 9.08e-05^a^ | 0.36^c^ | - | - | 8.42e-04^c^ | 6.22e-04^c^ | 7.91e-04^c^ |
